# Evolution and implementation of radiographic response criteria in neuro-oncology

**DOI:** 10.1093/noajnl/vdad118

**Published:** 2023-09-13

**Authors:** Divya Ramakrishnan, Marc von Reppert, Mark Krycia, Matthew Sala, Sabine Mueller, Sanjay Aneja, Ali Nabavizadeh, Norbert Galldiks, Philipp Lohmann, Cyrus Raji, Ichiro Ikuta, Fatima Memon, Brent D Weinberg, Mariam S Aboian

**Affiliations:** Department of Radiology and Biomedical Imaging, Yale School of Medicine, New Haven, Connecticut, USA; Department of Radiology and Biomedical Imaging, Yale School of Medicine, New Haven, Connecticut, USA; Department of Radiology and Biomedical Imaging, Yale School of Medicine, New Haven, Connecticut, USA; Department of Radiology and Biomedical Imaging, Yale School of Medicine, New Haven, Connecticut, USA; Tulane University School of Medicine, New Orleans, Louisiana, USA; Department of Neurology, Neurosurgery, and Pediatrics, University of California San Francisco, San Francisco, California, USA; Department of Radiology and Biomedical Imaging, Yale School of Medicine, New Haven, Connecticut, USA; Department of Radiology, University of Pennsylvania School of Medicine, Philadelphia, Pennsylvania, USA; Department of Neurology, Faculty of Medicine and University Hospital Cologne, University of Cologne, Cologne, Germany; Institute of Neuroscience and Medicine (INM-3), Research Center Juelich, Juelich, Germany; Center for Integrated Oncology (CIO), Universities of Aachen, Bonn, Cologne, and Duesseldorf, Cologne, Germany; Institute of Neuroscience and Medicine (INM-4), Research Center Juelich, Juelich, Germany; Department of Radiology, Washington University in St. Louis School of Medicine, St. Louis, Missouri, USA; Department of Radiology, Mayo Clinic, Phoenix, Arizona, USA; Department of Radiology and Biomedical Imaging, Yale School of Medicine, New Haven, Connecticut, USA; Department of Radiology, Emory University School of Medicine, Atlanta, Georgia, USA; Department of Radiology and Biomedical Imaging, Yale School of Medicine, New Haven, Connecticut, USA

**Keywords:** BT-RADS, RANO, RAPNO, Response Assessment, Volumetrics

## Abstract

Radiographic response assessment in neuro-oncology is critical in clinical practice and trials. Conventional criteria, such as the MacDonald and response assessment in neuro-oncology (RANO) criteria, rely on bidimensional (2D) measurements of a single tumor cross-section. Although RANO criteria are established for response assessment in clinical trials, there is a critical need to address the complexity of brain tumor treatment response with multiple new approaches being proposed. These include volumetric analysis of tumor compartments, structured MRI reporting systems like the Brain Tumor Reporting and Data System, and standardized approaches to advanced imaging techniques to distinguish tumor response from treatment effects. In this review, we discuss the strengths and limitations of different neuro-oncology response criteria and summarize current research findings on the role of novel response methods in neuro-oncology clinical trials and practice.

**Key Points**

- Response criteria rely on 2D measures, which are prone to interrater variability.
- Volumetrics and BT-RADS may offer a more comprehensive response assessment.
- Advanced MR techniques can differentiate tumor response from treatment effects.

Treatment response in neuro-oncology can be assessed using a variety of approaches that incorporate imaging, histopathology, molecular analysis, and functional assessment of patients.^1^ However, radiographic response, primarily on MRI, is often the main focus in clinical practice and trials. While response criteria have evolved over the years, selecting the best criteria that apply to multiple scenarios proves a challenge. The evaluation of bevacizumab for glioblastoma treatment in the early 2010s underscores this point.^1^ Two phase III clinical trials, AVAglio,^2^ and RTOG 0825,^3^ both found an increase in progression-free survival (PFS) of bevacizumab-treated glioblastoma patients with no difference in overall survival (OS). The AVAglio trial reported an improvement in patient-reported outcomes during the PFS period in the bevacizumab group.^2^ In contrast, the RTOG 0825 trial found that the bevacizumab group experienced a significant decline in patient-reported outcomes and cognitive performance at the end of the PFS period.^3^ Retrospective analysis of trial outcomes revealed a stark difference in radiographic response criteria used to define tumor progression^1^: AVAglio used RANO criteria, which account for nonenhancing tumor progression, while RTOG 0825 used MacDonald criteria, which only measure enhancing tumor. It is proposed that the AVAglio trial detected tumor progression earlier because its response assessment criteria included estimation of progression of nonenhancing disease. On the other hand, RTOG 0825 excluded nonenhancing disease from analysis and is presumed to have detected disease progression later leading to patients displaying clinical signs of progression before radiographic progression was measured.^1^

Radiographic response criteria have evolved over time to accommodate different tumor subtypes, patient populations, and treatment modalities.^4^ A comprehensive understanding of the differences in criteria is essential for proper clinical interpretation and decision-making. In this article, we review the evolution of current neuro-oncology response criteria (Figure 1), provide a summary of important radiographic criteria (Table 1), and discuss novel methods of response assessment, such as volumetric and advanced imaging techniques, including MR perfusion and spectroscopy.

**Table 1.** Summary of Key Response Criteria in Neuro-Oncology

Response Criteria	Tumor Type	Measurable Disease	Nonmeasurable Disease	Imaging Criteria
MacDonald (1990)	Glioblastoma	Not defined	Not defined	**CR**: disappearance of enhancing lesions and new lesions **PR:** ≥50% ↓ in enhancing lesions and no new lesions **SD:** <50% ↓ and <25% ↑ in enhancing lesions and no new lesions **PD:** ≥25% ↑ in enhancing lesions or appearance of any new lesion *CR and PR must be confirmed on ≥ *4-week follow-up*
RANO (2010)	High-grade glioma	T1PG: – both diameters ≥ 10 mm – seen on ≥ 2 slices	T1PG: – one diameter <10 mm – seen on <2 slices – cystic/necrotic tumor	**CR:** disappearance of enhancing lesions (measurable/nonmeasurable), stable/improved T2/FLAIR lesions, and no new lesions **PR:** ≥50% ↓ in measurable enhancing lesions, no progression of nonmeasurable disease, stable/improved T2/FLAIR lesions, and no new lesions **SD:** <50% ↓ and <25% ↑ in enhancing lesions, stable T2/FLAIR lesions, and no new lesions **PD:** ≥25% ↑ in enhancing lesions, significant ↑ in T2/FLAIR lesions, or appearance of any new lesion *CR and PR must be confirmed on ≥*4-week follow-up*
RANO-LGG (2011)	Low-grade glioma	T2/FLAIR: – both diameters ≥10 mm – seen on ≥2 slices	T2/FLAIR: – one diameter <10 mm – seen on <2 slices – cystic/necrotic tumor	**CR:** disappearance of all T2/FLAIR lesions and no new or ↑ enhancement **PR:** ≥50% ↓ in T2/FLAIR lesions and no new or ↑ enhancement **MinR:** ≥25% but <50% ↓ in T2/FLAIR lesions and no new or ↑ enhancement **SD:** <25% ↓ and <25% ↑ in T2/FLAIR lesions and no new or ↑ enhancement **PD:** ≥25% ↑ in T2/FLAIR lesions or any new lesion *PR must be confirmed on ≥*4-week follow-up*
iRANO (2015)	High-grade glioma post immunotherapy	T1PG: – both diameters ≥10 mm – seen on ≥2 slices	T1PG: – one diameter <10 mm – seen on <2 slices – cystic/necrotic tumor	**CR:** disappearance of enhancing lesions (measurable/nonmeasurable), stable/improved T2/FLAIR lesions, and no new lesions **PR:** ≥50% ↓ in measurable enhancing lesions, no progression of nonmeasurable disease, stable/improved T2/FLAIR lesions, and no new lesions **SD:** <50% ↓ and <25% ↑ in enhancing lesions, stable T2/FLAIR lesions, and no new lesions **PD:** ≥25% ↑ in enhancing lesions, significant ↑ in T2/FLAIR lesions, or any new lesions >6 months after therapy or ≤6 months after therapy with confirmation on second follow-up scan after 3 months *CR and PR must be confirmed on ≥ *4-week follow-up*
RANO-BM (2015)	Brain metastases	T1PG: – longest diameter ≥10 mm and perpendicular ≥5 mm – seen on ≥2 slices Target lesions: up to 5 measurable lesions selected based on largest size	T1PG: – lesions <10 mm – nonreproducible borders – dural, bony, leptomeningeal metastases – cystic-only lesions All considered nontarget lesions	**CR:** disappearance of target/nontarget lesions and no new lesions **PR:** ≥30% ↓ in sum of largest diameters of target lesions compared to baseline, stable/improved nontarget lesions, and no new lesions **SD:** <30% ↓ in target lesions compared to baseline and <20% ↑ compared to nadir, stable/improved nontarget lesions, and no new lesions **PD:** ≥20% ↑ compared to nadir, progression of existing enhancing or T2/FLAIR lesions, or presence of new lesion* *Patients not on immunotherapy
Modified RANO (2017)	Glioblastoma	T1PG: – both diameters ≥10 mm – seen on ≥2 slices	T1PG: – one diameter <10 mm – seen on <2 slices – cystic/necrotic tumor	**CR:** disappearance of enhancing lesions (measurable/nonmeasurable) compared to postradiation baseline **PR:** ≥50% ↓ in measurable enhancing lesions compared to postradiation baseline and SD/PR/CR compared to first follow-up **SD:** <50% ↓ and <25% ↑ in enhancing lesions compared to postradiation baseline or SD/PR/CR compared to smaller of nadir or PsP scan **PD:** ≥25% ↑ in enhancing lesions compared to postradiation baseline and confirmed on second scan or ≥25% ↑ in enhancing lesions compared to PsP scan
RAPNO-HGG (2020)	Pediatric HGG	T1PG or T2/FLAIR*: – both diameters ≥10 mm – seen on ≥2 slices – cystic lesion included if inseparable from solid *if no enhancement at baseline	T1PG or T2/FLAIR: – one diameter <10 mm – seen on <2 slices – cystic lesion outside of solid lesion – diffuse leptomeningeal disease DWI: – areas of restricted diffusion	**CR:** disappearance of measurable/nonmeasurable disease on T1PG and T2/FLAIR and complete resolution of areas of restricted diffusion on DWI **PR:** ≥50% ↓ in measurable disease and decreased areas of restricted diffusion on DWI **MinR:** ≥25% but <50% ↓ in measurable disease and decreased areas of restricted diffusion on DWI **SD:** <25% ↓ and <25% ↑ in measurable disease **PD:** ≥25% ↑ in measurable disease, clear increase in nonmeasurable disease, development of or increase in areas of restricted diffusion, or appearance of any new lesion
RAPNO-LGG (2020)	Pediatric LGG	T2/FLAIR*: – visible in 3 standard planes – both diameters ≥10 mm in all 3 planes – cystic lesion included if inseparable from solid *T1PG may be used for anterior optic gliomas, NF-1 tumors, or spinal tumors	T2/FLAIR: – visible in <3 standard planes – one diameter <10 mm in any plane – cystic lesion outside of solid lesion – diffuse leptomeningeal disease	**CR:** disappearance of measurable/nonmeasurable disease on T1PG and T2/FLAIR compared to baseline or best response **MR:** ≥75% ↓ in measurable disease but not enough for CR **PR:** ≥50% but <75% ↓ in measurable disease **MinR:** ≥25% but <50% ↓ in measurable disease **SD:** <25% ↓ and <25% ↑ in measurable disease **PD:** ≥25% ↑ in measurable disease *Response recommended to be confirmed on consecutive scan
BT-RADS (2018)	Any posttreatment tumor	N/A	N/A	**0:** unable to categorize **1a:** improvement due to decreasing tumor burden **1b:** improvement due to medication effect **2:** no change **3a:** worsened imaging due to treatment effects **3b:** worsened imaging due to mix of treatment effects and tumor burden **3c:** worsened imaging due to increasing tumor burden **4:** worsened imaging highly suspicious for tumor progression *Considers T2/FLAIR and T1PG changes in the context of treatment modality and time post treatment but does not incorporate measurements

BM = brain metastases; BT-RADS = Brain Tumor Reporting and Data System; CR = complete response; DWI = diffusion-weighted imaging; HGG = high-grade glioma; LGG = low-grade glioma; MinR = minor response; MR = major response; PD = progressive disease; PR = partial response; PsP = pseudoprogression; RANO = response assessment in neuro-oncology; RAPNO = response assessment in pediatric neuro-oncology; SD = stable disease; T1PG = T1 post-gadolinium.

**Figure 1.**
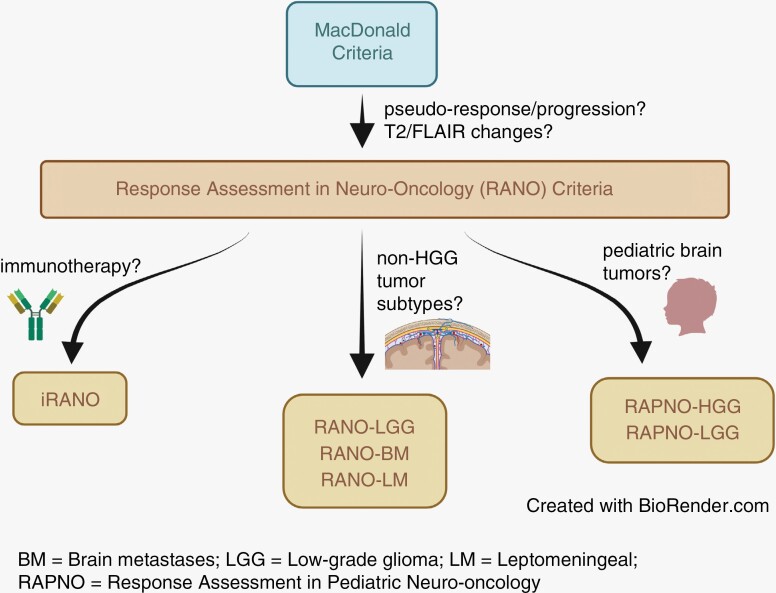
Evolution of response criteria in neuro-oncology.

## Bidimensional Response Criteria

### MacDonald Criteria

Developed in the late 20th century, the MacDonald criteria comprised the first attempt to objectively classify treatment response and were initially used on postcontrast CT images.^5^ Response was determined based on the percent change in the product of the maximal perpendicular diameters of enhancing tumor. However, the MacDonald criteria had two primary limitations: (1) failure to account for changes in nonenhancing tumor, often prevalent in slow-growing tumors, and (2) lack of guidelines to differentiate true disease progression/response from treatment-related phenomena, such as pseudoprogression and pseudoresponse.^5^

**Figure 2.**
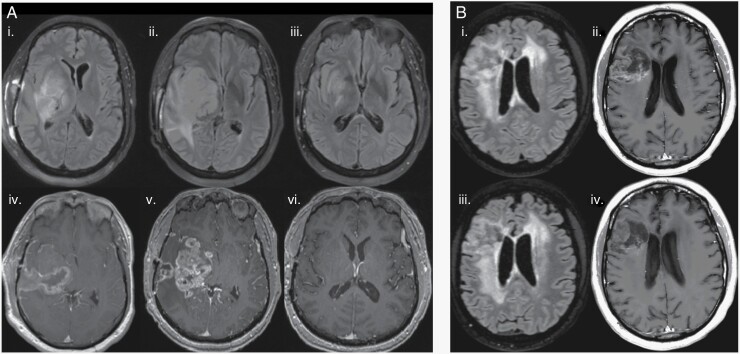
Treatment-related radiographic changes. (A) Pseudoprogression in a 50-year-old IDH wild-type diffuse midline glioma patient before initiating Pembrolizumab showing axial FLAIR (i) and T1 postcontrast (iv) images. Twenty days after Pembrolizumab therapy, there is an increase in vasogenic edema on axial FLAIR (ii) and contrast enhancement on T1 postcontrast (v) concerning for true progression versus pseudoprogression. Two months after Pembrolizumab therapy, there is a significant decrease in FLAIR hyperintensity (iii) and contrast enhancement (vi) confirming the previous diagnosis as pseudoprogression. (B) Pseudoresponse in a 55-year-old IDH wild-type glioblastoma patient before initiating bevacizumab (Avastin) showing axial FLAIR (i) and T1 postcontrast (ii) images. Two months after Avastin therapy, FLAIR hyperintensity and mass effect are unchanged (iii), but there was interval decrease in tumor enhancement on T1 postcontrast (iv).

Pseudoprogression is defined as an increase in tumor enhancement on the contrast enhanced T1-weighted sequence (T1 postcontrast) associated with initiating new therapy, including radiation or immunotherapy (Figure 2A). While the increase in tumor enhancement may coincide with functional changes in the individual, it is not reflective of a true increase in disease burden and often resolves within months without any treatment change.^6^ Pseudoresponse is defined by a decrease in tumor enhancement after starting treatment with antiangiogenic agents (eg, VEGF inhibitor) that is not reflective of a true decrease in disease burden. The decrease in tumor enhancement is likely caused by a transient decrease in permeability of the blood–brain barrier due to inhibition of abnormal vessel proliferation by antiangiogenic agents. Thus, signal intensity changes on T2/FLAIR sequences are often not present in pseudoresponse (Figure 2B).^1,7^

### Response Assessment in Neuro-Oncology Criteria

Response assessment in neuro-oncology (RANO) criteria were first developed to assess treatment response in high-grade glioma (HGG) and addressed the limitations in the MacDonald criteria. RANO criteria categorize disease into: (1) measurable—any lesion on T1 postcontrast with both diameters ≥10 mm and seen on ≥2 slices and (2) nonmeasurable—any lesion on T1 postcontrast where at least one diameter is <10 mm, is seen on <2 slices, or has cystic/necrotic components. RANO also considers nonenhancing T2/FLAIR changes, corticosteroid use, and clinical outcomes to classify an individual’s treatment response status.^8,9^ For example, according to RANO criteria, an individual would be considered to have progressive disease with a significant increase in T2/FLAIR hyperintensity regardless of the change in enhancing lesion. While RANO criteria provide numerical thresholds for assessing change in enhancing tumor, T2/FLAIR hyperintensity is characterized by overall visual assessment. To be classified as having response, the RANO criteria require all 3 of the following: a stable or decreased T2/FLAIR hyperintensity pattern, decreased to no dose of corticosteroids, and a stable or improved clinical status. The RANO working group also created specific guidelines for evaluating therapy-related phenomena. For example, increase in tumor enhancement after radiotherapy must be reevaluated after 12 weeks to differentiate pseudoprogression from true disease progression. Any additional areas of enhancement that arise during this 12-week period cannot be characterized as true disease progression unless they occur outside the target of radiation or are confirmed with pathology.^1^

The original RANO criteria made significant improvements over the MacDonald criteria, illustrated by one meta-analysis of 91 glioblastoma trials showing higher correlation between median PFS and OS using RANO criteria (*R*^2^ = 0.96, *n* = 8 trials) versus MacDonald criteria (*R*^2^ = 0.70, *n* = 83 trials).^10^ However, it became evident that the original RANO criteria were not entirely suitable for many other tumor subtypes. Three examples of RANO adaptations include iRANO, RANO-BM, and RANO-LGG.

The immunotherapy-RANO (iRANO) criteria were developed to evaluate treatment response in tumors specifically treated with immunotherapy, widely ranging from PD-L1 checkpoint inhibitors to chimeric antigen receptor T-cell therapy, after which therapy-related phenomena may obfuscate true changes in disease burden.^11^ iRANO criteria primarily address this challenge by accounting for the time between immunotherapy and posttreatment imaging. To distinguish true disease progression from pseudoprogression, iRANO recommends a repeat scan after 3 months to evaluate potential radiological progression detected on a scan taken less than 6 months after immunotherapy in the absence of clinical deterioration. Evaluation guidelines are also tied to specific recommendations about continuation or discontinuation of immunotherapy though initial iRANO criteria do not necessarily take into account other potential immunotherapy side effects like hypophysitis.^11^

Response assessment in neuro-oncology-BM criteria are used for brain metastases and include 3 major modifications: (1) classification of metastases into target and nontarget lesions for posttreatment response assessment; (2) quantification of overall target lesion burden; and (3) a bicompartmental approach to evaluating response.^12^ RANO-BM modifies RANO definitions of measurable and nonmeasurable disease. Measurable disease is defined as any metastatic lesion on T1 postcontrast with the longest diameter ≥10 mm and perpendicular diameter of ≥5 mm and is seen on ≥2 slices. Nonmeasurable disease is defined as any metastatic lesion on T1 postcontrast that is <10 mm, has nonreproducible borders, is in the dura, bone, or leptomeninges, or has only cystic components. Up to 5 measurable lesions are considered as target lesions based on size, measurement reliability, or active recurrence after local treatment. All nonmeasurable lesions are considered as nontarget. Overall response assessment is based on quantitative assessment of change in bidimensional (2D) measurements of target lesions and qualitative assessment of nontarget lesions. RANO-BM also employs a bicompartmental approach to response based on presence of both intra- and extracranial disease burden. Peritumoral edema is not included in the RANO-BM treatment response assessment, although treatment with steroids or immunotherapy can significantly change the appearance of peritumoral edema.

Response assessment in neuro-oncology-LGG criteria are used for low-grade glioma assessment and include 3 major modifications that are specific to LGG: (1) focus on T2/FLAIR changes; (2) creation of a “Minor Response” category; and (3) emphasis on clinical status in determining treatment response. Given the variable enhancement pattern and slow-growing nature of LGG, RANO-LGG primarily relies on changes in T2/FLAIR over those found on T1 postcontrast.^13^ Measurable and nonmeasurable diseases follow the same criteria as RANO, except that they are both assessed on T2/FLAIR. Since changes in T2/FLAIR hyperintensity of LGG are often small, RANO-LGG includes a “Minor Response” category to differentiate degree of response. One big challenge present in assessing response of LGG is correlating subtle changes on T2/FLAIR with clinical status. To address this challenge, RANO-LGG emphasizes integration of clinical endpoints, such as seizure frequency, vision, and cognitive performance, with radiographic changes to assess response.

### Modified Response Assessment in Neuro-Oncology Criteria

The modified Response Assessment in Neuro-oncology (mRANO) criteria were developed in response to some of the challenges encountered with the conventional RANO criteria, such as high interrater variability in qualitative assessment of T2/FLAIR changes and the impact of novel therapeutics, such as antiangiogenic agents and radiotherapy, on enhancing disease.^14^ There were 2 primary modifications in the mRANO compared to the original RANO criteria: (1) use of postradiotherapy scan as the baseline instead of the postsurgical scan and (2) response assessment solely based on measurable enhancing disease (excluding assessment of T2/FLAIR changes).^14^ The postradiotherapy scan is used as baseline since the timing of the postsurgical scan may vary greatly between patients and often contains postoperative artifacts, such as increased edematous changes, that can confound subsequent response assessment.^14^

Modified response assessment in neuro-oncology also outlines specific criteria for pseudoprogression and pseudoresponse using the postradiotherapy scan as baseline. For example, the initial posttreatment scan is compared to the baseline postradiotherapy scan. If there is at least a 25% increase in the bidimensional area of the lesion, the posttreatment scan is categorized as preliminary progression. The second posttreatment scan is then compared to the first posttreatment scan to determine if the preliminary progression is confirmed or whether it is pseudoprogression. If there is at least a 25% increase in the second post treatment scan compared to the first, then the scan shows confirmed progression. If not, the scan is categorized as pseudoprogression, and the third posttreatment scan is evaluated to determine whether there is confirmed progression or stable disease. Similarly, if the first posttreatment scan shows at least a 50% decrease in bidimensional tumor area compared to postradiotherapy baseline, it is categorized as preliminary response. It is classified as durable response if the second posttreatment scan shows stable or decreased tumor area compared to the first one. However, if it shows at least a 25% increase compared to the first posttreatment scan, it is then categorized as preliminary progression. There are slightly different variations of the mRANO criteria for recurrent compared to newly diagnosed glioblastoma.^14^

A recent study compared the RANO, mRANO, and iRANO criteria for PFS and OS of a large sample of newly diagnosed and recurrent glioblastomas.^15^ The study found a similar correlation between OS and PFS using RANO and mRANO in both new and recurrent glioblastomas. Interestingly, there was also no significant difference in correlation between PFS and OS using the RANO, mRANO, or iRANO criteria in immunotherapy-treated patients.^15^ However, there was a significant increase in correlation between PFS and OS in patients when the postradiotherapy scan was used as baseline as recommended by the mRANO criteria. In addition, there was an improvement in correlation between PFS and OS in newly diagnosed glioblastoma patients when a 12-week confirmation scan was used to diagnose progressive disease after initiation of radiotherapy. Surprisingly, evaluation of nonenhancing disease on FLAIR sequence did not lead to significantly better correlation between PFS and OS when comparing RANO to mRANO.^15^ While the FLAIR sequence is critical for neuroradiologic assessment of glioblastomas in clinical practice, this study showed that qualitative evaluation of the FLAIR sequence in a trial setting did not lead to improved correlation between PFS and OS. Thus, future research is needed on the role of measurement-based assessment of nonenhancing glioblastoma on FLAIR sequence and its correlation with PFS and OS.

The RANO 2.0 criteria^16^ have been proposed based on findings from the study comparing RANO, mRANO, and iRANO criteria in newly diagnosed and recurrent glioblastomas.^15^ The 3 main changes proposed by RANO 2.0 include the following: (1) use of the postradiotherapy scan as baseline; (2) confirmation of progressive disease that is found within 3 months after radiotherapy with a consecutive follow-up taken at 4–8 weeks; and (3) evaluation of IDH wild-type tumors with contrast enhancement without consideration of nonenhancing changes. Moreover, RANO 2.0 recommends application of these revised guidelines to all adult gliomas regardless of grade.^16^

### Response Assessment in Pediatric Neuro-Oncology Criteria

**Figure 3.**
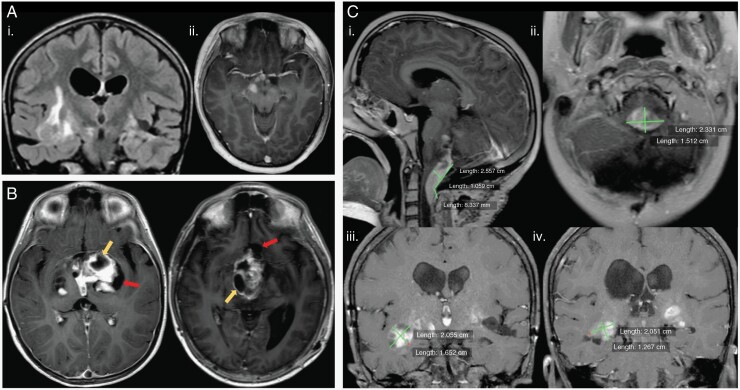
Pediatric low-grade gliomas. (A) Heterogeneous tumors with coronal FLAIR (i) illustrating an irregular hyperintense mass in the right temporal lobe and temporal stem and axial T1 postcontrast (ii) showing another lesion with irregular and ill-defined enhancement in the cerebral peduncle and along the optic tracts and optic chiasm. (B) Cystic components of pLGG illustrated on axial T1 postcontrast images with differentiation of tumor cysts (yellow arrows) and nontumor cysts (red arrows) according to RAPNO criteria. (C) Bidimensional measurement variability in pLGG. Images (i and ii) illustrate different bidimensional measurements made on a tumor in the foramen magnum on sagittal T1 postcontrast (i) and axial T1 postcontrast (ii). Images (iii and iv) are from a different patient with an infiltrative tumor in the bilateral temporal lobes. Coronal T1 postcontrast images with an enhancing lesion marked by one reviewer (iii) may vary significantly from measurements made on a different coronal T1 postcontrast image (iv) by a different reader.

Pediatric low-grade gliomas (pLGG) are the most common primary pediatric brain tumors and occur in the hypothalamus/optic chiasm (40%), cerebellum (25%), cerebral hemispheres (17%), and brainstem (9%). Like adult low-grade gliomas, pLGGs also have infiltrative and irregular borders, making bidimensional response assessment challenging (Figure 3A).^17,18^ In addition, pLGGs often have variable enhancement pattern and cystic components, which add to the heterogeneity of the tumor.^19^ The response assessment in pediatric neuro-oncology (RAPNO) criteria were adapted specifically to address these challenges.^20,21^ RAPNO focuses on nonenhancing infiltrative disease, which is best evaluated on T2/FLAIR sequences, similar to the RANO-LGG criteria. It also provides specific guidelines about including cystic components in bidimensional or 3 perpendicular plane tumor measurements. Cysts are differentiated into tumor and nontumor cysts, and only tumor cysts are included with the solid tumor in 2D measurements.^20^ A tumor cyst is defined as a cyst intermixed with solid portions of tumor and often surrounded by a ring of enhancement on T1 postcontrast.^20^ In contrast, a nontumor cyst is defined as a cyst located at the interface between tumor and healthy brain tissue that is clearly isolatable from the solid tumor portion (Figure 3B). RAPNO-LGG modifies the RANO definition of measurable disease to include lesions on T2/FLAIR that are visualized on all three standard planes, have both diameters ≥ 10 mm on all three planes, and include tumor cysts. Minor response criteria are also incorporated to account for the slow growth of pediatric LGGs. For more comprehensive response assessment, the RAPNO working group also incorporated guidelines on the use of standardized sequences and imaging protocols for tumor measurement. As many pLGGs occur in the optic chiasm, RAPNO criteria recommend correlation of radiographic changes to vision status when determining response in these tumors. In addition to RAPNO-LGG, there are many other RAPNO criteria, including those for HGG,^22^ diffuse intrinsic pontine glioma,^23^ craniopharyngioma,^24^ ependymoma,^25^ and medulloblastoma/leptomeningeal seeding.^26^

## Moving Beyond Bidimensional Methods of Response Assessment

### Role of Volumetrics

While bidimensional or 3 perpendicular plane tumor measurements are often the standard approach in clinical practice for response assessment, there are multiple limitations to this approach. One is interreader reliability when choosing the tumor cross-section with maximal perpendicular diameters, with one study finding only a moderate agreement (weighted kappa = 0.30) between site and central imaging review of posttreatment glioblastoma response using RANO criteria.^27^ This limitation is particularly apparent when measuring diffuse, irregularly shaped tumors, such as low-grade gliomas (Figure 3C).^18,28^

It has been proposed that measurement of tumor volume may provide a more comprehensive assessment of tumor burden, but accuracy of tumor volume measurements is dependent on thin slice isotropic image acquisitions (eg, T1 MPRAGE, SPGR, etc.), which have become standard of care in most hospitals only recently. Over the last few years, there has been an increase in research on the role of volumetrics in neuro-oncology, particularly in response assessment of glioblastomas.^29–34^ However, results on the utility of volumetrics remain mixed. One study of bevacizumab-treated glioblastoma did not show a significant improvement in posttreatment prognosis with volumetric measurements compared to 2D.^29^ In this study, both volumetric and 2D measurements of the enhancing tumor were performed, and participants were classified as having progressive disease based on changes in both volume and tumor area at 6 weeks posttreatment. There was a significant difference in OS between progressive disease and nonprogressive disease participants based on both volumetric and 2D methods of response.^29^ This study used a volume-extrapolated threshold of 40% for classifying progressive disease based on volumetric change, while the standard RANO threshold of 25% was used with 2D change. While this threshold was successful in differentiation of treatment progression from stable disease, evidence-based identification of optimal thresholds to stratify patients has not been performed to our knowledge. Another study by the same group compared RANO and different volumetric criteria in 148 participants with recurrent glioblastoma treated with bevacizumab from the BELOB prospective trial.^35^ The volumetric methods used to assess progressive disease at 6 and 12 weeks post treatment initiation included enhancing volume on T1 postcontrast, enhancing volume on digital subtraction imaging (T1 postcontrast minus T1 precontrast), and enhancing volume on T1 postcontrast or digital subtraction imaging plus nonenhancing volume on FLAIR. Although the hazard ratio for death was highest at both follow-up times when progressive disease was measured by 2D RANO criteria, there was no significant difference in hazard ratios between RANO and volumetric methods.^35^ It is important to note that this study used the volume-extrapolated threshold of 40% when classifying progressive disease based on enhancing or subtraction volumes and a threshold of 25% based on nonenhancing volume. This highlights the importance of empirically validating volumetric thresholds, as results may differ based on which thresholds are applied to specific tumor volumes.^29,35^

Meanwhile, a study on the role of volumetrics in adult LGG found a significant increase in progression-free survival based on volumetric compared to 2D methods.^36^ The study also revealed that volumetrics had less interreader discordance and more stable growth rates than 2D, suggesting that volumetrics may be a more reliable and consistent method of response assessment in adult LGG.^36^ Despite preliminary promise of volumetrics to offer a more comprehensive assessment of treatment response, it is still not considered a standard part of response assessment by the RANO working group and is largely limited to the research setting. Further research is warranted to evaluate clinical utility of volumetrics, particularly among various tumor types.

In addition to the applicability of volumetrics to different tumor types, significant research is under way for methods of incorporation of tumor volumetrics into clinical practice, which would make this method more available to practices outside of highly specialized central imaging review laboratories that are mostly focused on clinical trials (M. von Reppert, unpublished data, 2022; D. Ramakrishnan, unpublished data, 2023). The evaluation of the role of using standardized isotropic thin slice sequences within protocols for treatment response assessment is also under way. Optimization and standardization of image acquisition, increasing availability of measurement tools, and assessing the role of volumetrics to assess different tumor subcompartments will pave the way for establishing volumetrics in treatment response assessment.

Limitations of volumetrics include its time intensity and lack of readily available tools in clinical practice.^37^ A rise of AI-based automated segmentation tools can address these challenges.^38–40^ For example, incorporation of segmentation algorithms and volumetric tools directly into the picture archiving and communication system (PACS) can facilitate clinical practice workflows.^41^ Furthermore, PACS-based tools to automatically track lesion growth can make response assessment more reliable and efficient.^38^ One study showed that interrater agreement for time to progression was significantly greater with an AI-based tool for tracking longitudinal tumor volume changes compared to manual 2D measurements using the RANO criteria.^38^ It is also important to consider algorithm generalizability, which can be limited by the diversity of training and validation datasets.^42^ Nevertheless, increasing efforts to make data sets publicly available will contribute to algorithm development.^43^

### Brain Tumor Reporting and Data System (BT-RADS)

**Figure 4.**
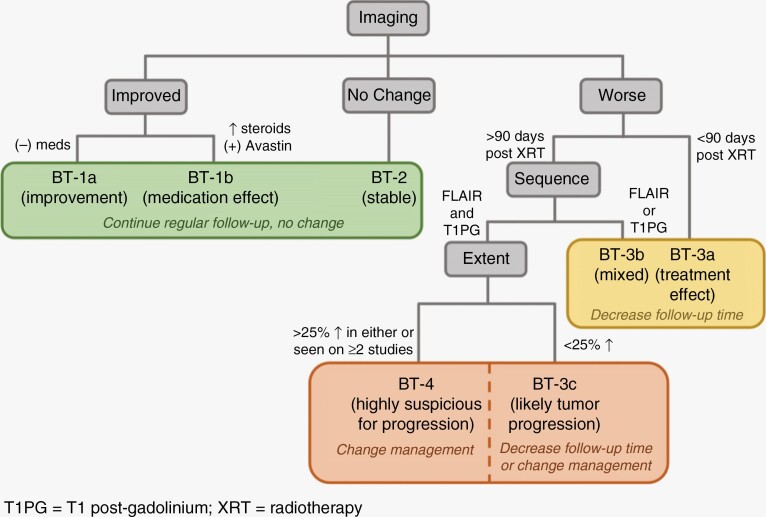
BT-RADS response assessment flowchart.

**Figure 5.**
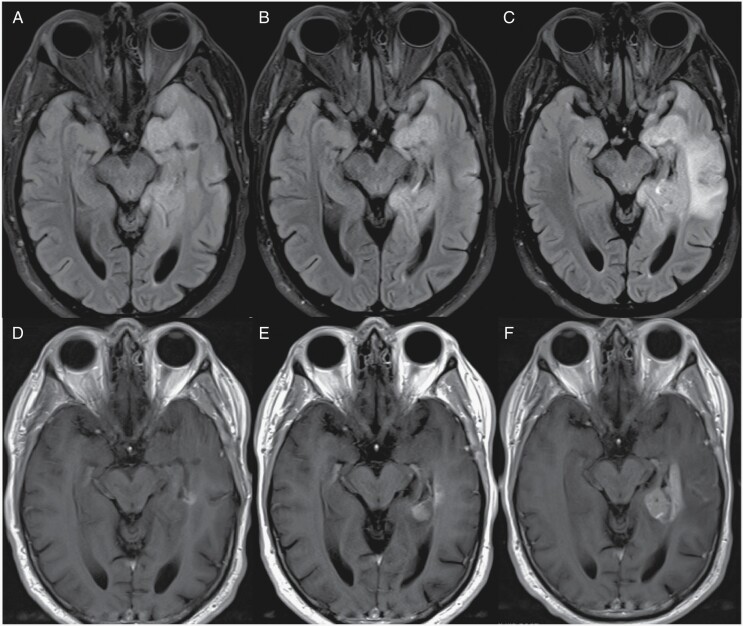
BT-RADS criteria used for response assessment of a WHO Grade 2, IDH wild-type astrocytoma. Axial FLAIR and T1 postcontrast images at 7 months (A, D), 10 months (B, E), and 13 months (C, F) after surgical resection are shown. At 10 months, a score of 3c (recommendation for decreased interval of follow-up) was assigned given worsening enhancement (E) and marginally increased FLAIR hyperintensity (B). At 13 months, a score of 4 (recommendation to change clinical management) was assigned given further worsening of both enhancement (F) and FLAIR hyperintensity (C) over multiple studies.

The previously described response criteria rely heavily on measurements, which may be prone to interreader variability, and are only part of the assessment that brain tumor imaging undergoes in clinical radiology reporting. In addition, these criteria rely on predefined thresholds of change in tumor measurements, which may not necessarily lead to changes in clinical management. The BT-RADS criteria were designed to assess treatment response by neuroradiologists in a standardized and structured way with each response category tied to a specific recommendation in clinical management (Figure 4). BT-RADS were developed by an interdisciplinary team of neuroradiologists, neurosurgeons, and neuro-oncologists in response to survey feedback from clinicians (27 radiologists and 26 nonradiologists) about the quality of MRI reports for brain tumors at an academic institution.^44^ The BT-RADS framework was designed to be incorporated into clinical radiology reports, with criteria focused on visual assessment of FLAIR changes, enhancement, and tumor mass effect in the context of treatment initiation without emphasis on quantitative measurements as is used in RANO response criteria. A score of 0–4 is assigned based on neuroradiologist’s assessment of tumor and treatment effects, and each score includes a recommendation about frequency of follow-up given the likelihood of tumor response or progression (Figure 5).

Overall, the BT-RADS criteria have shown promise in improving the quality of MRI reports and clinical decision-making. One study evaluating the use of BT-RADS to describe response in posttreatment gliomas revealed that reports using the BT-RADS structure were more succinct, definitive, and incorporated more clinical history.^45^ Another study looking at the relationship between survival and BT-RADS scores in HGGs found a 53% increase in the risk of death with every one-point increase in BT-RADS score.^46^ The results from these studies show that BT-RADS offers standardized guidelines that increase the utility of MRI reports, have good correlation with prognosis, and can improve clinical management.

### Advanced Imaging Techniques

Many advanced magnetic resonance (MR) imaging techniques can help with treatment response assessment and differentiation of tumor progression from therapy-related phenomena.^47^ One widely used technique is MR perfusion, which reveals information about tumor blood flow and volume. Various techniques for obtaining MR perfusion include dynamic susceptibility contrast (DSC) and dynamic contrast enhanced (DCE) imaging, which use an injected contrast agent, and arterial spin labeling (ASL), which has the added benefit of avoiding gadolinium contrast agents. MR perfusion can help differentiate tumor recurrence from radiation necrosis, as areas of tumor typically have higher blood flow, blood volume, and permeability. Perfusion MRI can also evaluate nonenhancing tumor that can serve as complementary analysis to FLAIR sequences.^48,49^ One of the major limitations of perfusion is susceptibility artifact from blood products in the posttreatment setting, which can significantly reduce sensitivity of DSC imaging for recurrent tumor. Furthermore, there is poor reproducibility of threshold values due to lack of standardization in perfusion-weighted imaging protocols and postprocessing pipelines.^50^ Significant efforts in the standardization of imaging protocols and postprocessing pipelines are being performed as part of the Open Science Initiative for Perfusion Imaging (OSIPI).^51^

**Figure 6.**
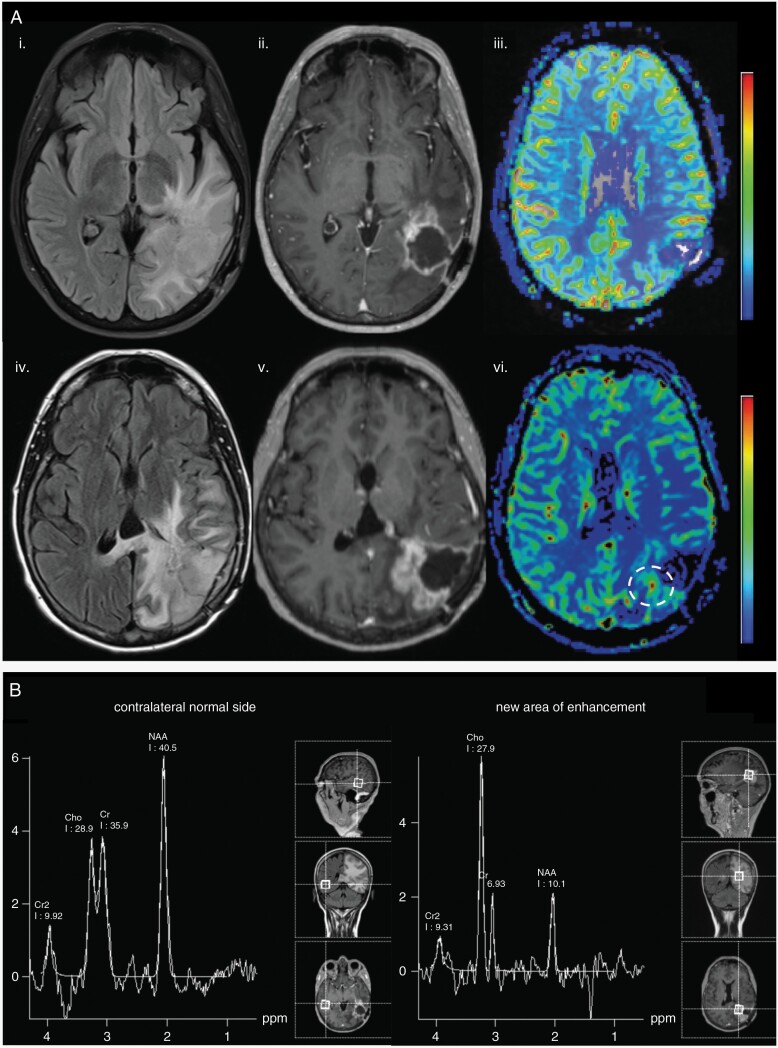
Advanced imaging techniques to distinguish tumor recurrence from radiation necrosis in a 59-year-old glioblastoma patient 4 years after initial resection. (A) Baseline images (i–iii) showing FLAIR (i), T1 postcontrast (ii), and DSC cerebral blood volume (CBV) maps (iii). Subsequent follow-up 2 months post repeat resection (iv–vi) shows increased FLAIR hyperintensity (iv), increasing thickness of T1 postcontrast enhancement surrounding the resection cavity (v), and increase in CBV on DSC perfusion map (white circle, vi). These findings are suspicious for tumor recurrence. (B) MR spectroscopy results of the same patient reveal a Cho to NAA ratio >2 in the region of enhancement (right) compared to contralateral normal side (left), further supporting a diagnosis of tumor recurrence.

Another advanced imaging technique is MR spectroscopy, which detects tumor chemical metabolites. These metabolites serve as biomarkers of tumor processes in both pretreatment and posttreatment states.^52,53^ Two such metabolites are *N*-acetylaspartate (NAA), a marker of neuronal integrity, and choline (Cho), a marker of cell membrane turnover. Radiation necrosis and recurrent tumors often present as enhancing areas on MRI and can be difficult to differentiate (Figure 6A). MR spectroscopy may show the presence of lipid peaks, which indicate presence of necrosis. However, an elevated Cho to NAA ratio indicates more cellular turnover and is more suggestive of the presence of viable proliferating tumor in a treated region (Figure 6B), especially if the ratio is greater than or equal to 2.^54^ MR spectroscopy is particularly useful for diagnosing subtypes of pediatric brain tumors, which often have more heterogeneity in tumor cell types and biological metabolites compared to adult brain tumors. In fact, the relative ratio of NAA, creatine, Cho, and lactate metabolites can be used to differentiate between posterior fossa tumor subtypes in pediatric patients.^55^ One of the major limitations of MRS is the lack of reimbursement for clinical use in the United States and nonstandardized protocols with a time-intensive process to position the measurement voxel within suspected regions of the tumor.^56^ To address this limitation, whole-brain MRS protocols and multivoxel protocols have been developed.^57^

MR diffusion works based on tracking the motion of water molecules within tissue. It typically provides a diffusion-weighted image (DWI) and computationally derived apparent diffusion coefficient maps (ADC). Areas of high intensity on DWI and corresponding low ADC values indicate areas of decreased water movement, mostly seen in infarcted tissue. However, low ADC values can also identify areas of increased cellular density or neoplasia in some CNS tumors^58^ and can favor areas of recurrent glioblastoma in the setting of bevacizumab therapy.^59^ MR diffusion can also be used to characterize and differentiate brain lesions.^60,61^ One of the limitations of using DWI in treatment response assessment is the susceptibility artifact due to blood products, which will lead to low ADC values.

Finally, PET has demonstrated tremendous potential for treatment response assessment in primary and metastatic tumors.^62^ FDG PET is commonly used in the United States to differentiate posttreatment changes from tumor recurrence, but is significantly limited due to high background signal from normal FDG uptake in the brain. On the other hand, amino acid PET has been demonstrated to have significant advantages in delineation of tumor margins, detection of residual tumor after resection, and assessment of posttreatment changes from tumor recurrence in both primary and metastatic brain tumors.^62–66^ Various ^18^F-labeled amino acid PET tracers are used across the world, with the most common tracers being ^18^F-FET, ^18^F-FDOPA, and the newly emerging tracer in neuro-oncology, ^18^F-Fluciclovine.^67–71^^11^C-Methionine is one of the earliest used amino acid PET tracers but has not been widely used due to the short half-life of ^11^C and the requirement for a cyclotron on site.^72,73^ PET RANO has been involved in addressing these criteria and establishing the guidelines for using PET in treatment response assessment.^64^ While advanced imaging methods provide significant promise in adding clinical value in treatment response assessment of brain tumors, standardization of protocols and development of validation studies are still needed prior to incorporating these methods into response criteria.

### Assessment of Brain Health

One of the major limitations of current radiographic treatment response criteria is the focus on evaluation of tumor and its imaging characteristics with limited assessment of nontumor brain parenchyma. RANO criteria assess patient-related outcomes in addition to history of corticosteroid use, which allows evaluation of nontumor effects of treatment. However, evaluation of nontumor brain parenchyma is also critical for assessment of treatment response because most cancer therapies have significant toxicity that impact patient morbidity and mortality. Knowledge of these off-target effects can guide the therapy decision process. Several MRI biomarkers are available to evaluate the off-target effects of chemotherapy within the CNS, including white matter damage on FLAIR sequence, formation of microbleeds on susceptibility weighted images, and volume loss on isotropic T1 precontrast images. These biomarkers have been shown to correlate with cognitive outcomes and may be used to assess brain health in patients with cognitive decline.^74–80^ Future research is needed to elucidate how these metrics and biomarkers of treatment-related early CNS damage can be incorporated into response assessment methods.

## Concluding Remarks

Clinical decision-making in neuro-oncology is often based on accurate radiographic response assessment of tumors, which commonly uses subjective assessment and bidirectional or 3 perpendicular plane tumor measurements that are subject to intra- and interrater variability. While the MacDonald criteria were the first objective criteria in neuro-oncology, the RANO criteria addressed several of the limitations present in the MacDonald criteria and are currently the most widely used response criteria in the field. Efforts have been made by the RANO working group to modify the criteria for specific tumor subgroups and treatment modalities. The results of these efforts include criteria such as RANO-BM, RANO-LGG, iRANO, RAPNO-LGG, and RAPNO-HGG, among several others.

Over the last few years, alternative methods to conventional bidimensional criteria have been proposed to assess treatment response more comprehensively. At the forefront of this field has been volumetrics, which shows promise to address the limitations of 2D methods in slow-growing tumors with irregular borders. However, further research is required to study the role of volumetrics in different tumor subgroups. In addition, the advent of fully automated segmentation tools that incorporate artificial intelligence algorithms may decrease the inter- and intrarater variability of manual volumetric segmentations. The BT-RADS method has been developed for clinical implementation in imaging reports, with response categories tied to specific recommendations for clinical management. The criteria are largely based on neuroradiologist’s visual assessment of tumor in multiple planes and do not emphasize numerical thresholds to define response. Early studies on BT-RADS have shown promise in improving quality of MRI reports and leading to more definitive clinical management, but more research on the use of this assessment criteria and how it performs in the setting of multimodal imaging is needed.

Finally, advanced MR and PET imaging techniques can provide information beyond conventional MRI, such as differentiation of tumor recurrence from treatment-related changes within the treated tumor and assessment of damage to the normal surrounding brain parenchyma. Moreover, there has been a significant increase in understanding of tumor interaction with surrounding normal brain and off-target effects of chemotherapy on normal brain parenchyma. Incorporation of brain health metrics into response criteria has the potential to provide critical information to clinicians for patient management. Given the strengths and limitations of different response criteria, a multimodal approach, which considers varying tumor biology, patient characteristics, and evolving treatments, is essential for accurate response assessment in neuro-oncology.
